# Malaria Situation in the Peruvian Amazon during the COVID-19 Pandemic

**DOI:** 10.4269/ajtmh.20-0889

**Published:** 2020-09-03

**Authors:** Katherine Torres, Freddy Alava, Verónica Soto-Calle, Alejandro Llanos-Cuentas, Hugo Rodriguez, Lidia Llacsahuanga, Dionicia Gamboa, Joseph Vinetz

**Affiliations:** 1 Laboratorios de Investigacion y Desarrollo, Facultad de Ciencias y Filosofia, Universidad Peruana Cayetano Heredia, Lima, Peru;; 2 Instituto de Medicina Tropical Alexander von Humboldt, Universidad Peruana Cayetano Heredia, Lima, Peru;; 3 Laboratorio Amazonía-ICEMR y Enfermedades Emergentes Sede Iquitos, Universidad Peruana Cayetano Heredia, Lima, Peru;; 4 Dirección Regional de Salud Loreto (DIRESA), Iquitos, Perú;; 5 Dirección de Prevención y Control de Enfermedades Metaxénicas y Zoonosis, Ministerio de Salud, Lima, Perú;; 6 Universidad Nacional de la Amazonía Peruana, Iquitos, Perú;; 7 Departamento de Ciencias Celulares y Moleculares, Facultad de Ciencias y Filosofia, Universidad Peruana Cayetano Heredia, Lima, Peru;; 8 Section of Infectious Diseases, Department of Internal Medicine, Yale School of Medicine, New Haven, Connecticut

## Abstract

The Peruvian Ministry of Health reports a near absence of malaria cases in the Amazon region during the COVID-19 pandemic. However, the rapid increase in SARS-CoV-2 infections has overwhelmed the Peruvian health system, leading to national panic and closure of public medical facilities, casting doubt on how accurately malaria cases’ numbers reflect reality. In the Amazon region of Loreto, where malaria cases are concentrated, COVID-19 has led to near-complete closure of the primary healthcare system, and diagnosis and treatment of acute febrile illnesses, including malaria, has plummeted. Here, we describe the potential association of COVID-19 with a markedly reduced number of reported malaria cases due to the reduced control activities carried out by the Peruvian Malaria Zero Program, which could lead to malaria resurgence and an excess of morbidity and mortality.

Malaria continues to be a high-priority public health concern in tropical and subtropical regions of the world,^1^ including the Peruvian Amazon, which is also endemic for several other acute febrile diseases such as dengue and leptospirosis. In Peru, malaria is concentrated primarily in the Loreto department, where transmission is maintained in rural and peri-urban communities.

In 2017, the Peruvian government brought a formal malaria elimination plan into law, the Malaria Zero Program (MZP), that takes a community-level approach to control malaria with the goal of elimination by 2030. The MZP has three complementary and partially overlapping phases: 1) the control phase, testing and treating, which focuses on the elimination of symptomatic infections and has a duration of 3 years; 2) a first-phase (elimination phase), the goal of which is to eliminate malaria parasites from individuals at a regional level by targeting asymptomatic and low-parasite-density infections (in addition to symptomatic infections) for an extended span of 10 years; and 3) a final elimination phase, to identify and ameliorate residual malaria transmission foci, including reintroductions which is expected to last 15 years.^2,3^ The MZP is currently in the first (control) phase prioritized in the high endemicity region of Loreto. Since 2018, the number of malaria cases has decreased significantly (Figure 1), a reduction of 50% from 2017 to 2019. This reduction has been attributed to work carried out in Loreto, such as test-and-treat strategies, provision of free antimalarials under control of the Ministry of Health (MoH), and larviciding and pyrethroid spraying.^4^

**Figure 1.**
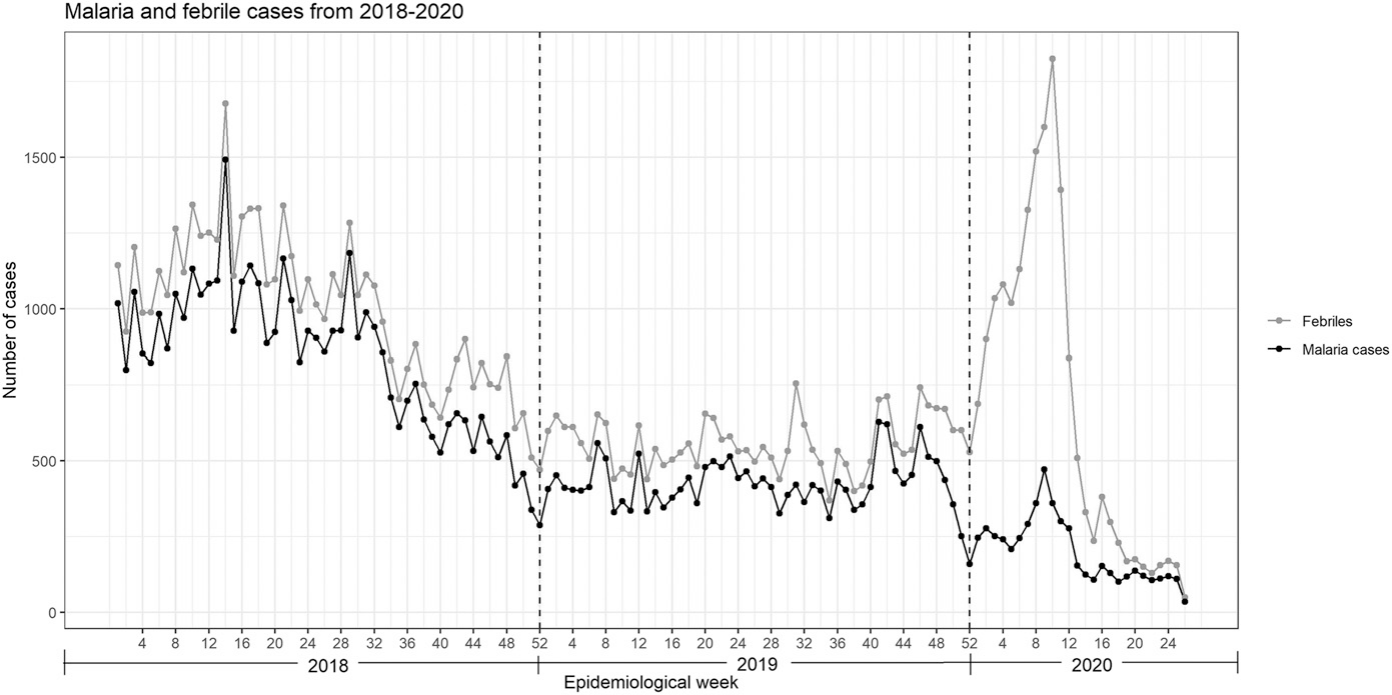
Malaria and febrile cases from 2018 to 2020 in the Loreto department of Peru. Source: Centro de Prevención y Control de Enfermedades–CPC de la Dirección Regional de Salud (DIRESA) de Loreto–MINSA. Data were extracted directly from NOTIV (data server from CPC-DIRESA in Iquitos). Updated at epidemiological week 27, 2020. COVID-19 febrile cases are not included. Cases reported in the first 3 months of the year; after that, the number decreased significantly. Dengue outbreak from December 2019 to March 2020.

Malaria control activities in Peru take place primarily in areas with a high number of indigenous communities which are most affected by malaria. Activities focus on active case detection and include the delivery of malaria rapid diagnostic tests and antimalarial drugs to trained health promoters, who are key to diagnosing and treating uncomplicated malaria cases in their communities. Another important malaria control activity in the Loreto region is based on the use of insecticide-impregnated mosquito nets for vector control. Community health promoters also conduct educational activities in communities, another part of malaria control programs. All of these activities are routinely carried out by each institution that provides health services in malaria-endemic areas.^2,3,5^ These activities are strengthened by the MZP, which has an assigned budget for malaria control in areas of very high and high risk of malaria transmission. All of these organized activities require in-person work and contact, which make them vulnerable to interruptions by external events such as COVID-19.

The health system in the Loreto region is structured as health networks (Redes) and, within them, micro-networks (MicroRedes), where each MicroRed has a number of health posts as the unit providing basic health care. Each health post is required to report the number of malaria cases every week to the Directorate of Health of Loreto.

When COVID-19 started to spread throughout Peru, the Peruvian government established strict quarantine measures and a nationwide lockdown in March 2020. Despite the relatively quick response, the pandemic continued to spread, hitting the Loreto region particularly hard. In the face of the current obvious urgency of the COVID-19 pandemic impacting a region with many other acute febrile illnesses, we aimed to gain insight about possible associations of the pandemic with the malaria situation in the Loreto region of the Peruvian Amazon.

During the first months of lockdown in Peru, the rapid increase in SARS-CoV-2 infections destabilized the Peruvian health system in manifold ways, especially in the capital of the Loreto region, Iquitos. The urgency and rapid increase in COVID-19 cases in Iquitos (Table 1),^6^ the lack of evidence-based clinical protocols, the lack of personal protective equipment (PPE), the lack of oxygen, and, in some way, frank panic at all hospitals throughout the country have exacerbated the public health crisis. This crisis was prominent at all hospitals in Iquitos—which became dedicated COVID-19 hospitals—because healthcare workers lacked PPE, leading to many becoming ill from COVID-19, and some even dying. Hence, MoH-supported facilities (health posts and hospitals) have closed for all clinical activities, except for COVID-19. These closures have contributed to delays in reporting malaria testing and confirmed cases in the entire region. On the other hand, people at home were also affected in diverse ways, many seemed to be dominated by fear, especially of attending health facilities. All of this has limited the collecting of key data, which hampers the determination of whether COVID-19 may have negative effects on national programs and research after 4 months of lockdown, and after that critical situation in Iquitos.

**Table 1:** COVID-19 cases in Loreto region, Peru

Province	District	Population	Confirmed cases	Suspected cases	Annual incidence rate × 10,000 Hab*	Deaths
Maynas	Napo	17,043	553	1,025	925.89	3
	Indiana	11,830	162	89	212.17	20
	Iquitos	157,591	1,721	1,215	186.31	724
	Punchana	95,426	736	412	120.30	278
	Mazan	14,424	85	31	80.42	4
	Belen	79,260	338	242	73.18	175
	San Juan	161,997	612	432	64.45	327
	Fernando Lores	21,173	96	6	48.17	5
	Alto Nanay	2,912	9	0	30.91	0
	Las Amazonas	10,345	14	6	19.33	2
	Torres Causana	5,388	0	2	3.71	1
Alto Amazonas	Yurimaguas	75,576	844	246	144.23	64
	Teniente Cesar Lopez	6,889	10	6	23.23	–
	Lagunas	14,976	25	3	18.70	–
	Balsa Puerto	18,244	7	3	5.48	–
Datem del Marañon	Barranca	14,239	236	219	319.54	–
	Pastaza	6,655	4	14	27.05	–
	Manseriche	10,854	5	12	15.66	–
	Andoas	12,936	0	3	2.32	–
	Cahuapanas	8,719	0	2	2.29	–
Loreto	Nauta	31,500	522	95	195.87	28
	Trompeteros	11,247	107	3	97.80	7
	Tigre	8,811	76	0	86.26	–
	Parinari	7,596	40	3	56.61	–
	Urarinas	15,399	10	1	7.14	–
Putumayo	Putumayo	4,418	44	145	427.80	1
Ramon Castilla	Ramon Castilla	25,275	601	8	240.95	26
	Yavari	16,367	175	54	139.92	10
	Pebas	17,859	72	55	71.11	4
	San Pablo	16,821	64	0	38.05	8
Requena	Jenaro Herrera	5,888	11	32	73.03	4
	Requena	31,574	112	94	65.24	28
	Yaquerana	3,124	9	0	28.81	–
	Puinahua	6,289	11	1	19.08	–
	Maquia	8,760	10	1	12.56	5
	Emilio San Martin	7,830	2	4	7.66	4
	Capelo	4,655	0	2	4.30	1
	Sapuena	5,151	0	2	3.88	2
Ucayali	Contamana	28,552	363	16	132.74	27
	Sarayacu	17,343	62	1	36.33	5
	Padre Marquez	7,947	21	0	26.43	–
	Vargas Guerra	9,349	24	0	25.67	4
	Inahuaya	2,782	4	0	14.38	1
	Pampa Hermosa	11,124	2	1	2.70	–

Centro de Prevención y Control de Enfermedades–CPC de la Dirección Regional de Salud de Loreto–MINSA. Updated at epidemiological week 26, 2020. Cases are considered since the onset of symptoms.

At first sight, available data and observations seem to be limited by the availability of routine surveillance and basic implementation strategies in Loreto communities, which are usually carried out as part of regular activities of the MoH and supplemented by MZP and funded research projects. Regular interventions carried out by the MZP plummeted from the second half of April, when COVID-19 cases began their exponential increase in Loreto. Other malaria control activities, such as vector control, active detection, and community education, were postponed because of COVID-19, although from February 10 to February 28, the intra-household recycling activities with residual insecticide were reported for 656 houses in communities around the Iquitos-Nauta road between the 15th and the 23rd km, protecting 1,815 people. According to MZP preliminary reports, the number of malaria-specific interventions and activities to date in 2020 has dropped by at least 60% compared with the same period in 2019. This situation may well continue until regional authorities organize the new way to do routine public health interventions under the COVID-19 situation. In addition, from 414 health posts, only 80% have been able to comply with the weekly notification in recent reporting periods, likely because of the COVID-19 situation. Thus, the health post malaria notification rate decreased to 62% during the pandemic lockdown. Nevertheless, recently, the malaria regional database has experienced some variability in the quality of information provided. Importantly, malaria is likely to resurge in the region in the absence of malaria diagnosis and treatment, leading to increased mortality and morbidity.

The apparent decline observed on the number of malaria cases since the pandemic started is remarkable. The reduction from February to March was 20%; from March to April, 44%; from April to May 21, 88%; and from May to June 21, 99%, ultimately reaching as few as 35 malaria cases in the last week of June. These reductions are striking for the region’s known high transmission season (January–May). Moreover, the decrease in cases in Loreto after the outbreak was not only reported for malaria but also for dengue and leptospirosis—other major causes of acute undifferentiated fever—the highest number of cases reported before the pandemic (Table 2).

**Table 2:** Malaria, dengue, and leptospirosis cases from 2018 to 2020

		Years		
		2018	2019	2020
Number of febrile cases		51,945	29,013	17,493*
Number of cases of malaria	*Plasmodium falciparum*	9,340	3,891	851†
	*Plasmodium vivax*	34,198	18,034	4,310†
Number of cases of dengue		2,177	2,522	6,882‡
Number of cases of leptospirosis		1,020	3,032	5,422†

Centro de Prevención y Control de Enfermedades–CPC de la Dirección Regional de Salud de Loreto–MINSA. Updated at epidemiological week 27, 2020.

* COVID-19 febrile cases are not included.

† Cases reported in the first 3 months of the year; after that, the number decreased significantly.

‡ Dengue outbreak from December 2019 to March 2020.

The precise reasons why this COVID-19 situation may be associated with problems with malaria control cannot be fully determined at this time. Several factors likely contribute to an apparent decrease in cases. In addition to administrative shutdown, it is likely that people are not going to health facilities to get diagnosed (in case they do not have a health promoter in their communities) because the health centers are closed, or because of the convenience of feeling safe taking just an antipyretic at home and avoiding the risk of getting COVID-19. Furthermore, quarantine has compelled people to stay at home and limited their access to public transportation.

Research projects have also been affected at many institutions after the government declared the lockdown. Universities and research institutions had to completely suspend all laboratory and fieldwork activities. Consequently, research studies on diseases other than COVID-19 were paused. Closing laboratories and stopping research studies challenge the collaborative relationship between government and academia, so this situation could be a setback on the way to eliminate malaria. This situation is particularly worrisome because previous attempts to eliminate malaria in the region suggest that without focused control, a rapid bounce back effect can take place even after years of efforts.^5^ However, the full association of COVID-19 with this unprecedented situation of malaria control has yet to be determined.

Not only will this pandemic challenge our advances in malaria control in the Loreto region of Peru but it will also affect the way we conduct research and interventions from now on. Because these activities cannot be indefinitely halted, they must be restarted following appropriate biosafety measures. New biosecurity strategies should be implemented, specifically the use of PPEs to protect us from the hazards of field and laboratory work, especially when we have to take samples, because of the high rate of COVID-19 infections in this area.

In Iquitos, COVID-19 cases (symptomatic and asymptomatic) are being controlled (Figure 2), but expansion remains of high concern, especially after the suspension of the lockdown in Peru, which obligates the MoH to strengthen the strategies to control not only COVID-19 but also other endemic diseases in the Loreto region.

**Figure 2.**
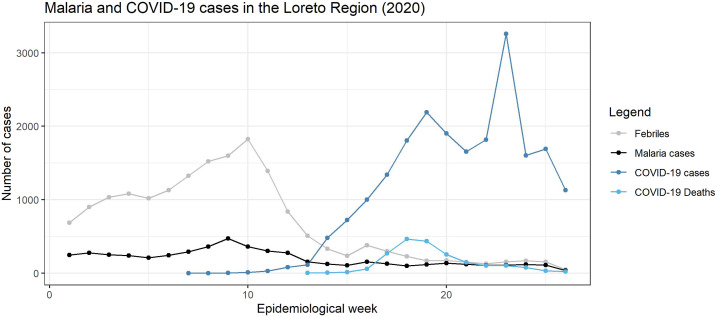
Malaria and COVID-19 cases in the Loreto region (2020). Source: Centro de Prevención y Control de Enfermedades–CPC de la Dirección Regional de Salud de Loreto–MINSA.

The COVID-19 pandemic is likely to have direct, measurable impacts on patients with endemic tropical and neglected infectious diseases and on related research. Here, we have described an apparent associate of COVID-19 with reduction in malaria control activities as carried out by the Peruvian MZP. We now confront the challenge of delivering malaria-related health care in remote areas where COVID-19 cases increase, especially affecting endangered indigenous communities. National and regional health systems remain challenged to provide adequate quality health care for those vulnerable, affected populations. The effect of COVID-19 on malaria resurgence and excess morbidity and mortality due to malaria can only be determined by prospective surveillance; however, this may be accomplished.
